# PERIOPERATIVE CARE IN DIGESTIVE SURGERY: THE ERAS AND ACERTO PROTOCOLS - BRAZILIAN COLLEGE OF DIGESTIVE SURGERY POSITION PAPER

**DOI:** 10.1590/0102-672020240001e1794

**Published:** 2024-05-06

**Authors:** José Eduardo de AGUILAR-NASCIMENTO, Ulysses RIBEIRO, Pedro Eder PORTARI-FILHO, Alberto Bicudo SALOMÃO, Cervantes CAPOROSSI, Ramiro COLLEONI, Dan Linetzky WAITZBERG, Antonio Carlos Ligocki CAMPOS

**Affiliations:** 1 Centro Universitário de Varzea Grande, Department of Surgery - Varzea Grande (MT), Brazil; 2 Universidade de São Paulo, Faculty of Medicine, Department of Gastroenterology - São Paulo (SP), Brazil;; 3 Universidade Federal do Rio de Janeiro, Faculty of Medicine - Rio de Janeiro (RJ), Brazil; 4 Universidade Federal de São Paulo, Escola Paulista de Medicina, Department of Surgery - São Paulo (SP), Brazil; 5 Universidade Federal do Paraná, Faculty of Medicine, Department of Surgery - Curitiba (PR), Brazil.

**Keywords:** Perioperative Care, Nutrition Therapy, Crystalloid Solutions, Guideline, Assistência Perioperatória, Terapia Nutricional, Soluções Cristaloides, Guia

## Abstract

**BACKGROUND:**

The concept introduced by protocols of enhanced recovery after surgery modifies perioperative traditional care in digestive surgery. The integration of these modern recommendations components during the perioperative period is of great importance to ensure fewer postoperative complications, reduced length of hospital stay, and decreased surgical costs.

**AIMS:**

To emphasize the most important points of a multimodal perioperative care protocol.

**METHODS:**

Careful analysis of each recommendation of both ERAS and ACERTO protocols, justifying their inclusion in the multimodal care recommended for digestive surgery patients.

**RESULTS:**

Enhanced recovery programs (ERPs) such as ERAS and ACERTO protocols are a cornerstone in modern perioperative care. Nutritional therapy is fundamental in digestive surgery, and thus, both preoperative and postoperative nutrition care are key to ensuring fewer postoperative complications and reducing the length of hospital stay. The concept of prehabilitation is another key element in ERPs. The handling of crystalloid fluids in a perfect balance is vital. Fluid overload can delay the recovery of patients and increase postoperative complications. Abbreviation of preoperative fasting for two hours before anesthesia is now accepted by various guidelines of both surgical and anesthesiology societies. Combined with early postoperative refeeding, these prescriptions are not only safe but can also enhance the recovery of patients undergoing digestive procedures.

**CONCLUSIONS:**

This position paper from the Brazilian College of Digestive Surgery strongly emphasizes that the implementation of ERPs in digestive surgery represents a paradigm shift in perioperative care, transcending traditional practices and embracing an intelligent approach to patient well-being.

## RESUMO

**RACIONAL:**

O conceito introduzido pelos protocolos de recuperação após a cirurgia modifica os cuidados perioperatórios tradicionais em cirurgia digestiva. A integração desses componentes modernos de recomendações, durante o período perioperatório, é de grande importância para garantir menos complicações pós-operatórias, redução do tempo de internação hospitalar e diminuição dos custos cirúrgicos.

**OBJETIVOS:**

Enfatizar os pontos mais importantes de um protocolo multimodal de cuidados perioperatórios.

**MÉTODOS:**

Análise criteriosa de cada recomendação dos protocolos ERAS e ACERTO, justificando sua inclusão no atendimento multimodal recomendado para pacientes de cirurgia digestiva.

**RESULTADOS:**

Os programas de recuperação avançada (PRAs), tais como os protocolos ERAS e ACERTO, são a base dos cuidados perioperatórios modernos. A terapia nutricional é de grande importância na cirurgia digestiva e, portanto, tanto os cuidados nutricionais pré-operatórios, quanto pós-operatórios são fundamentais para garantir menos complicações pós-operatórias e reduzir o tempo de internação hospitalar. O conceito de pré-habilitação é outro elemento-chave nos PRAs. O manuseio de fluidos cristalóides em perfeito equilíbrio é vital. A sobrecarga de fluidos pode atrasar a recuperação dos pacientes e aumentar as complicações pós-operatórias. A abreviação do jejum pré-operatório para duas horas antes da anestesia é agora aceita por diversas diretrizes das sociedades cirúrgicas e de anestesiologia. Combinadas com a realimentação pós-operatória precoce, essas prescrições não são apenas seguras, mas também podem melhorar a recuperação de pacientes submetidos a procedimentos digestivos.

**CONCLUSÕES:**

Este posicionamento do Colégio Brasileiro de Cirurgia Digestiva enfatiza fortemente que a implementação de PRAs em cirurgia digestive, representa uma mudança de paradigma no cuidado perioperatório, transcendendo as práticas tradicionais e adotando uma abordagem inteligente para o bem-estar do paciente.

## SUMMARY OF THE MAIN RECOMMENDATIONS:

- Provide comprehensive preoperative education to patients, discussing their expectations, and the importance of their active participation in their recovery.
- Emphasize the importance of preoperative nutrition, including immune nutrition and carbohydrate loading, to enhance energy reserves and support optimal recovery.
- Implement more restrictive fluid management strategies tailored to individual patient needs, avoiding excessive hydration and associated complications.
- Implement the use of symbiotics perioperatively.
- Discourage prolonged preoperative fasting and encourage the intake of clear fluids up to two hours before surgery to maintain hydration and energy levels.
- Implement a multimodal strategy to prevent nausea and vomiting.
- Utilize minimally invasive surgical techniques whenever feasible to minimize tissue trauma and accelerate recovery.
- Emphasize laparoscopic or robotic-assisted surgeries in conjunction with ERPs principles to optimize outcomes.
- Minimize opioid use in favor of multimodal analgesia, incorporating regional anesthesia, non-opioid medications, and patient-controlled analgesia.
- Employ a multimodal approach to pain management, combining various analgesic modalities to address pain from multiple angles.
- Encourage early postoperative mobilization to enhance recovery, reduce complications, and minimize the risk of thromboembolic events.
- Promote early initiation of oral or enteral nutrition to support gastrointestinal function and expedite recovery.
- Discourage prolonged postoperative fasting and encourage a prompt return to oral intake to prevent nutritional depletion.
- Initiate early discharge planning to facilitate a smooth transition from the hospital to home or a lower-level care facility.

## INTRODUCTION

During the last decade of the past century, some revision papers and international societies’ guidelines introduced the concept of fast-track surgery in the literature. This approach basically suggested modifications in traditional perioperative care to enhance recovery after surgery. The so-called fast-track protocol was mainly advocated by Kehlet in Europe and by Wilmore in the USA^33^. At the beginning of the current century, a group of surgeons and anesthesiologists from some Northern European countries introduced a comprehensive protocol, improving the fast-track approach.

**Figure 1 -:**
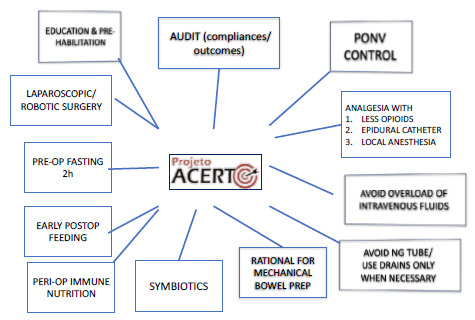
Elements of the Total Acceleration of Postoperative Recovery Project (ACERTO) protocol.

PONV: postoperative nausea and vomiting; Pre-op: preoperative; Postop: postoperative; Peri-op: preoperative; Bowel prep: bowel preparation; NG: nasogastric.

This evidence-based guideline for perioperative care was named ERAS (Enhanced Recovery After Surgery) protocol^9^ ^,^ ^31^ ^,^ ^34^ ^,^ ^35^ ^,^ ^40^ ^,^ ^43^ ^,^ ^56^. In 2005, a Brazilian multimodal protocol of perioperative care was initiated in a university hospital and first published in 2006^5^ ^,^ ^17^ ^,^ ^18^ ^,^ ^19^ ^,^ ^54^ (Figure 1).

In recent literature, the term enhanced recovery protocols (ERPs) has been frequently used to express multimodal perioperative care and this current position paper will, therefore, adopt it herein.

**Figure 2 -:**
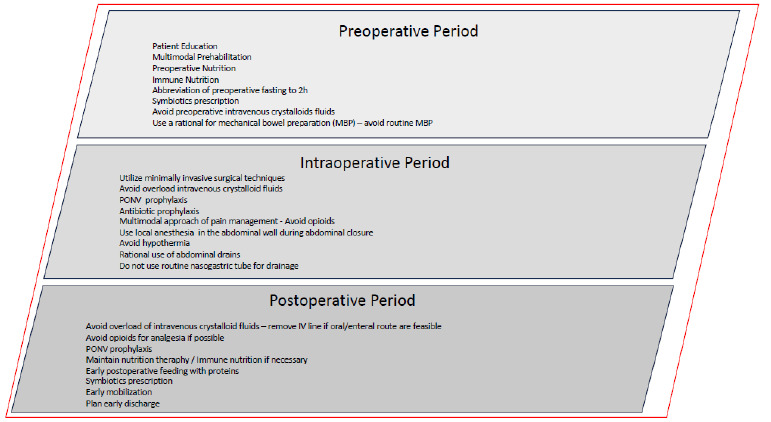
Perioperative care recommended by the Brazilian College of Digestive Surgery for digestive surgery.

ERPs involve a comprehensive approach to patient management before, during, and after surgery, integrating various strategies to enhance outcomes and accelerate recovery (Figure 2)^6^ ^,^ ^60^.

PONV: postoperative nausea and vomitin.)

This approach combines surgical technique with nutrition, pharmacological, psychological, and physiological interventions to address different aspects of the surgical process^15^ ^,^ ^44^ ^,^ ^53^. Key components include preoperative optimization^8^ ^,^ ^18^ ^,^ ^21^ ^,^ ^31^ ^,^ ^34^ ^,^ ^35^, which focuses on patient preparation and risk mitigation, intraoperative strategies such as balanced anesthesia and minimally invasive techniques, and postoperative care involving pain management^30^, early mobilization^62^, and nutrition^60^. Perioperative nutrition plays an important role in enhanced recovery protocols especially by improving the nutritional condition of the patient before and after surgery as well as reducing the fasting period^8^ ^,^ ^18^ ^,^ ^25^ ^,^ ^32^ ^,^ ^36^. Multimodal analgesia, a cornerstone of these protocols, utilizes a combination of analgesic agents to minimize reliance on opioids, thereby reducing side effects and promoting quicker recovery^17^ ^,^ ^30^ ^,^ ^31^. Psychosocial elements, like patient education and psychological support, play a crucial role in minimizing perioperative stress and improving overall well-being^5^ ^,^ ^19^ ^,^ ^35^ ^,^ ^36^.

The integration of these modern prescriptions during the perioperative period is fundamental to ensure fewer postoperative complications, reduce the length of hospital stay, and decrease surgical costs^2^ ^,^ ^6^ ^,^ ^45^ ^,^ ^56^ ^,^ ^57^.

Therefore, this knowledge should be enforced to increase the perceptions of surgeons^12^. This position paper aims to emphasize not only the most important points of an ERP but also recommend it in the perioperative care of digestive procedures.

### What is prehabilitation and how can it enhance the recovery of surgical patients?

Prehabilitation, a proactive and structured approach to preparing patients for surgery during the preoperative period, has gained prominence as an integral component of enhanced recovery protocols^28^. Rather than focusing solely on postoperative rehabilitation, prehabilitation aims to optimize a patient’s physical and psychological status before surgery, potentially leading to improved outcomes and enhanced recovery^14^.

Tailored exercise programs, encompassing aerobic conditioning, strength training, and flexibility for approximately four weeks before surgery, can improve overall fitness and enhance a patient’s physiological reserve^26^. This can be particularly beneficial for patients undergoing major elective surgeries. Preoperative anxiety and stress can impact postoperative recovery. Psychological interventions, such as counseling and stress-reducing techniques, are integral to prehabilitation to improve mental well-being^14^.

Prehabilitation often involves interventions to address modifiable risk factors, such as arterial hypertension and diabetes, besides ceasing smoking and alcohol consumption^27^. Alcohol andsmoking cessation, along with guidance on lifestyle modifications, contribute to a healthier preoperative state. Providing patients with information about the surgical process, expected outcomes, and the importance of their active participation in prehabilitation fosters a sense of empowerment. Informed and engaged patients may better adhere to prehabilitation plans^14^ ^,^ ^27^.

In summary, prehabilitation represents a proactive strategy to optimize a patient’s physical and mental health before surgery, aligned with the principles of enhanced recovery. Integrating prehabilitation into the preoperative period contributes to a more comprehensive and patient-centered approach to perioperative care^14^.

Currently, the term multimodal prehabilitation has been used, including nutrition as a key component. However, as can be seen below, we will look at perioperative nutrition separately^42^. A systematic review and meta-analysis showed that multimodal prehabilitation significantly decreased the length of hospital stay by two days in patients undergoing colorectal surgery^26^.

**Figure 3 -:**
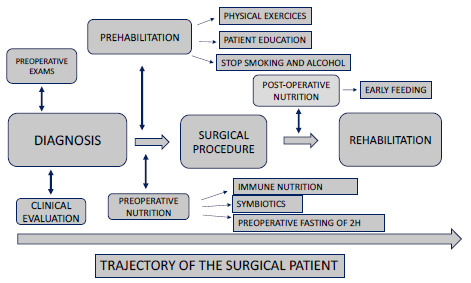
Surgical patient journey following a multimodal enhanced recovery protocol.

Therefore, we recommend multimodal prehabilitation to accelerate the functional capacity of surgical patients. The trajectory of patients following these recommendations can be seen in Figure 3.

### How preoperative nutrition attention and prescription can benefit surgical patients?

Adequate nutritional status approach before a moderate to major surgical procedure has been consistently regarded as highly important^44^ ^,^ ^60^. Therefore, we strongly suggest an assessment before surgery. The nutritional risk score (NRS-2002) followed by subjective global assessment are examples of tools to evaluate the nutritional status^19^ ^,^ ^60^.

Surgical patients who are candidates to digestive procedures, especially in oncology, have a high prevalence of malnutrition alone or associated with sarcopenia^44^. Adequate preoperative nutrition for at least 7-14 days before surgery can not only reduce postoperative complications but also decrease the length of hospital stay and costs^60^.

The Total Acceleration of Postoperative Recovery Project (ACERTO) suggests the immediate nutritional intervention (INTERNUTI) protocol that proposes the initiation of nutrition intervention immediately when the surgical procedure is decided^5^ ^,^ ^19^. The INTERNUTI protocol is relevant because there is usually an interval of several days or weeks, and also a gap between the surgical decision moment and the day of the surgery due to laboratory exams, schedule of the surgeon or surgical center, and other technicalities. More than 14 days of oral supplementation with proteins is not a problem!

Actually, implementing INTERNUTI is a better choice than deciding lately to postpone the procedure to initiate preoperative nutrition. The options for preoperative nutrition are oral supplementation, enteral feeding and if the digestive route is contraindicated or cannot be assessed, a parenteral nutrition, or even the association of the three modalities^44^. This approach is consistent with a large number of randomized trials and meta-analyses^60^.

These data provided strong evidence that preoperative nutrition decreases postoperative complications and length of stay.

Clinical trials in surgical patients have investigated the impact of immune-enhancing nutrition, including arginine and omega-3 fatty acids, on postoperative outcomes^1^ ^,^ ^32^ ^,^ ^36^ ^,^ ^56^ ^,^ ^60^. These studies suggest a reduction in infectious complications, including surgical site infections, in patients receiving these nutritional interventions.

The benefits, which also include reduction of nosocomial stay, can be observed with at least five days of immune-enhanced nutrition preoperatively in major procedures and continuing for seven days postoperatively in cases of severe malnutrition^59^.

### How to prescribe preoperative fasting for elective digestive procedures and which benefits can be associated with reducing preoperative fasting protocol?

Preoperative fasting, traditionally mandated for several hours before surgery, has undergone a paradigm shift with the adoption of ERPs such as the ERAS or ACERTO protocols^5^ ^,^ ^19^ ^,^ ^22^ ^,^ ^35^ ^,^ ^36^. The conventional approach aimed to reduce the risk of aspiration during anesthesia, but prolonged fasting^16^ can lead to various adverse effects, including dehydration, insulin resistance, and increased stress response. In recent years, there has been a shift toward a more liberalized preoperative fasting approach, allowing patients to consume clear fluids up to two hours before surgery^23^ ^,^ ^37^ ^,^ ^44^ ^,^ ^60^. This change is supported by growing evidence suggesting that a shorter fasting period combined with carbohydrate supplementation is safe (does not increase the risk of broncho aspiration)^46^ ^,^ ^50^, besides positively impacting patient outcomes^2^ ^,^ ^20^ ^,^ ^23^ ^,^ ^34^.

Protocols of various international societies of anesthesiologists recommend fasting of solids for 6-8 hours and allow 200-400 mL of clear fluids containing carbohydrate up to two hours before anesthesia^34^. Supplements, often in the form of clear liquids, containing easily digestible carbohydrates (12% maltodextrin, 200-400 mL given two hours before induction of anesthesia), help to maintain metabolic function and mitigate the catabolic effects of prolonged fasting^22^ ^,^ ^61^. These supplements given two hours before surgery contribute to improved insulin sensitivity, reduce muscle protein breakdown, and provide a readily available energy source for the body^17^ ^,^ ^18^ ^,^ ^19^ ^,^ ^20^ ^,^ ^31^ ^,^ ^34^ ^,^ ^35^ ^,^ ^36^.

This approach aligns with the principles of enhanced recovery, emphasizing the importance of maintaining physiological functions and minimizing the stress response to surgery^44^ ^,^ ^61^.

The combination of whey protein and carbohydrate supplementation administered three hours before the anesthesia induction has gained attention within the ACERTO protocol as a potential strategy to further optimize preoperative nutritional status and positively impact surgical outcomes^17^ ^,^ ^44^ ^,^ ^46^ ^,^ ^47^. Whey protein, the so-called fast protein, is easily digestible and absorbed, rich in essential amino acids, and has been recognized for its ability to stimulate protein synthesis and enhance muscle preservation. When combined with carbohydrates, this nutritional strategy is not only safe but also provides a dual benefit by supporting both protein anabolism and maintaining glycogen stores^3^ ^,^ ^46^ ^,^ ^47^.

Consuming whey protein and carbohydrates a few hours before anesthesia induction helps address the catabolic effects of surgery, providing the body with essential nutrients during the perioperative fasting period. This preoperative nutrition may contribute to improved muscle strength, reduced postoperative insulin resistance, and faster recovery^49^.

Although this abbreviation protocol of preoperative fasting can be used in most elective digestive surgeries, there are contraindications that need to be emphasized in cases as follows: gastroparesis, intestinal obstruction, ileus, use of semaglutide or anti-spasmodic drug, and in critically-ill patients^44^ ^,^ ^60^.

### What are the benefits of early postoperative feeding and how safe is it in patients with digestive anastomosis?

Traditionally, surgeons reinitiate oral/enteral diet in digestive surgery after ileus resolution. As a result, patients usually stay 2-4 days on a nil-per-mouth regimen and receive 2-3 L of intravenous (IV) crystalloid fluids per day^19^. Not rarely, during this early postoperative period, patients also receive a nasogastric tube to drain gastrointestinal contents.

Conversely, early postoperative feeding following abdominal surgery, even with a gastrointestinal anastomosis, is currently recognized to be safe and is considered a crucial component of all enhanced recovery protocols^5^ ^,^ ^6^ ^,^ ^15^ ^,^ ^31^ ^,^ ^35^ ^,^ ^36^ ^,^ ^44^. This change in postoperative refeeding management is based on contemporary consistent evidence that strongly supports the notion that initiating early oral/enteral feeding is safe and can also offer several advantages in the postoperative period^19^ ^,^ ^39^.

Feeding shortly after abdominal surgery even with anastomosis is believed to promote mucosal integrity, decrease ileus time, enhance the function of the gastrointestinal tract, decrease length of stay, and reduce the risk of postoperative complications^19^ ^,^ ^36^ ^,^ ^44^. Early enteral nutrition not only provides essential nutrients to support the body’s recovery but also helps maintaining the gut barrier function and modulate the inflammatory response.

Early postoperative feeding can be initiated even in the postoperative recovery room^24^ but is defined as the initiation of oral/enteral diet within 24 hours after surgery^60^. The timing and composition of postoperative feeding diet may vary based on the type of surgery and individual patient factors. The notion of gradual progression of the diet from clear fluids to a full diet lacks evidence, and nowadays, modern guidelines suggest that the progression of diet consistency should take into consideration the tolerance of the patient^18^ ^,^ ^35^ ^,^ ^36^ ^,^ ^39^ ^,^ ^44^.

Guidelines based on randomized trials have firmly shown that early postoperative feeding can lead to faster recovery of bowel function, reduced length of hospital stay, and improved patient satisfaction^18^ ^,^ ^31^ ^,^ ^35^ ^,^ ^46^. However, individual patient characteristics and the nature of the surgical procedure must be considered when determining the appropriate timing and composition of postoperative nutrition. For example, early enteral nutrition through either a nasojejunal tube or jejunostomy has much more evidence strength to be safe than early oral nutrition when esophageal resection followed by anastomosis is done. On the other hand, early postoperative oral feeding after colorectal surgery is suggested by almost all guidelines of surgical and nutritional societies^18^ ^,^ ^31^ ^,^ ^36^.

Finally, the early commencement with diets containing protein is more efficient than other diets and should be the one to be prescribed. A recent meta-analysis showed that an early postoperative diet with proteins may reduce mortality in colorectal surgery^52^.

### How to prescribe intravenous crystalloid fluids in uncomplicated digestive surgery?

The restrictive use of IV crystalloid fluids perioperatively has gained attention as a strategy to optimize fluid management and improve patient outcomes^7^. Traditional perioperative fluid practices often involve liberal administration of IV fluids; however, growing evidence suggests that a more conservative approach may be beneficial in certain patient populations and surgical scenarios.

Excessive administration of IV crystalloid fluids can lead to complications such as tissue edema, including pulmonary congestion, ileus, impaired organ function, and electrolyte imbalances^11^. A restrictive fluid strategy focused on tailoring fluid administration to individual patient needs, considering factors such as preoperative hydration status, type of surgery, and ongoing losses, is highly recommended. Studies indicated that a more restrictive fluid approach during surgery, particularly in patients without significant fluid deficits, may contribute to reduced postoperative complications, shorter hospital stays, and improved recovery^38^. By avoiding fluid overload, the risk of complications including respiratory compromise and impaired tissue oxygenation can be minimized. The evidence shows that a small amount of fluid overload causing small weight gain of around 1-2 kg does not have adverse effects, but when the body weight increases 2.5-3 kg (or more) due to fluid excess, adverse effects can be expected and the risk of complications increase^37^ ^,^ ^38^. Adverse effects of excess saline and its consequent hyperchloremic acidosis on postoperative outcome, anastomotic healing, and gastrointestinal function (ileus) have been evidenced^38^.

Findings in various studies and meta-analyses showed that salt and water retention is not a harmless and inevitable epiphenomenon, and should be avoided whenever possible by restricting maintenance fluids to the amount necessary to achieve a zero balance. It is important to note that the appropriateness of a restrictive fluid strategy depends on various factors, including patient comorbidities and the nature of the surgical procedure^51^. Close monitoring of hemodynamic parameters, combining crystalloid fluids with colloids when indicated, and individualized fluid management are essential components of this approach^41^.

The enhanced recovery protocols such as ERAS and ACERTO recommend no preoperative IV fluids in elective surgery when the patient is in good condition^20^ ^,^ ^31^. Shortening preoperative fasting time with clear fluids up to two hours before surgery may supply patients’ needs.

Patients undergoing minor surgeries such as inguinal herniorrhaphy and orificial anal procedures may not need IV fluids postoperatively because early oral commencement of diet and hydration is preferable^24^. Even videolaparoscopic cholecystectomy patients may recover well without IV fluids. The use of a salinized scalp to maintain IV access for IV drugs (such as anti-emetics, analgesics, and antibiotics) may help to enhance the recovery of patients compared to the maintenance of a bag of 500-1000 mL of crystalloid fluid to keep an effective IV access. However, in major procedures, IV crystalloid solutions are necessary but also could be stopped as soon as the patient receives oral hydration and diet.

**Figure 4 -:**
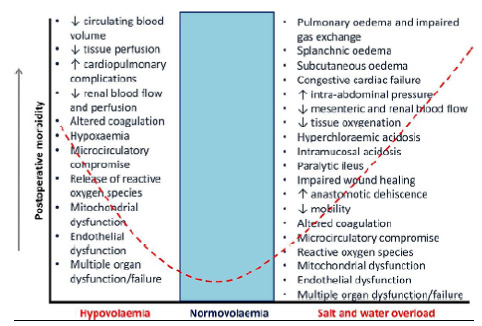
Volemia condition and postoperative morbidity^37^.

As mentioned above, early postoperative feeding and hydration are very relevant items in ERAS and ACERTO protocols^19^ ^,^ ^31^ ^,^ ^36^. The evidence points out that balanced solutions such as Ringer or Plasmalyte should be preferred over 0.9% saline or 5-10% dextrose solutions^19^ ^,^ ^38^ ^,^ ^48^ ^,^ ^58^. When necessary, the volume should be no more than 30 mL/kg/day under normal conditions. Colloid solutions may be combined to crystalloid solutions to reduce the total daily volume. An adequate daily hydric fluid balance should be done in major procedures. The concept of near-zero fluid balance is a cornerstone of the ERAS protocol^38^. Figure 4 shows the importance of fluid balance to reduce complications.

### Why is early postoperative mobilization recommended by enhanced recovery programs?

Early postoperative mobilization is a cornerstone of ERPs and plays a pivotal role in expediting recovery, reducing complications, and improving overall patient outcomes. ERPs emphasize initiating ambulation and mobilization as soon as possible after surgery, typically within the first 24 hours^19^ ^,^ ^35^ ^,^ ^36^. However, patients should be educated about this early postoperative mobilization before surgery because adherence may be low^29^. This preoperative counseling may increase adherence to early mobilization^19^ ^,^ ^29^ ^,^ ^40^ ^,^ ^56^.

This approach is applicable to a wide range of surgical procedures, including abdominal surgeries, joint replacements, and other interventions. However, as an isolated element to reduce the morbidity rate and duration of hospital stay, early mobilization alone failed to help enhanced recovery^62^ ^,^ ^63^. Then, this prescription should be associated with other elements of ERPs.

### Is the prophylaxis of postoperative nausea and vomiting included in enhanced recovery programs?

PONV prophylaxis is another crucial component of perioperative care. It can significantly impact a patient’s postoperative experience; therefore, preventing these symptoms aligns with the goals of ERPs to enhance recovery and improve patient outcomes^20^ ^,^ ^31^ ^,^ ^34^. An initial assessment of PONV risk can be done and the anesthesiologist should start a multimodal approach by IV drugs to mitigate the risk^13^ ^,^ ^31^. During the early postoperative period the use of ondansetron (4-8 mg, two or three times a day [bid or tds]) is more effective than metoclorpramide^55^ ^,^ ^61^ ^,^ ^64^.

ERPs also advocate for a more restrictive approach to fluid management, avoiding overhydration^19^ ^,^ ^34^. This is relevant to PONV prophylaxis, as excessive IV fluids can contribute to nausea and vomiting^38^. These modern protocols of perioperative care often recommend carbohydrate loading two hours before surgery and avoiding prolonged fasting^19^ ^,^ ^34^ ^,^ ^36^ ^,^ ^44^. Decreasing preoperative fasting can also contribute to a reduced risk of PONV.

### Is preoperative mechanical bowel preparation imperative in digestive surgery?

Most digestive procedures can be performed without preoperative mechanical bowel preparation. However, MBP has been subject of evolving practices and debates in colorectal surgery. Traditionally, MBP involved the use of laxatives and enemas to cleanse the bowel before surgery. Nevertheless, ERPs principles challenge the routine use of MBP and recommend a more selective approach based on individual patient and surgical factors^34^ ^,^ ^35^. MBP may be considered selectively for specific cases where there is a higher risk of infection or anastomotic leakage. This is the case of anastomosis involving the rectum. Despite that, the decision should be based on a careful evaluation of the benefits and risks, taking into account the individual patient characteristics and the surgical procedure^65^.

According to ACERTO protocol, MBP is not necessary before a right colectomy^20^. During preoperative nutrition, it is wise to recommend the patients to exclude fibers from the diet one week before the surgery.

### Is the use of symbiotics a valuable option during the perioperative period?

The use of symbiotics, which are a combination of probiotics and prebiotics, has been a topic of interest in the field of digestive surgery and is included in the ACERTO protocol^18^. Probiotics are live microorganisms that confer health benefits when administered in adequate amounts, while prebiotics are non-digestible compounds that promote the growth and activity of beneficial bacteria in the gut. When combined, they form symbiotics, aiming to positively influence the gut microbiota^10^.

A wide variety of surgical digestive diseases can cause dysbiosis. Then, a preoperative approach with symbiotics, theoretically, can confer a healthy environment in the gut microflora^50^. By enhancing the colonization of beneficial bacteria, symbiotics may help create an environment less favorable to pathogenic organisms. In accordance, randomized trials and meta-analysis showed that symbiotics can reduce postoperative complications and length of hospital stay^4^ ^,^ ^10^ ^,^ ^50^.

Although without evidence, in patients with compromised immune system, a careful evaluation before incorporating symbiotics into their perioperative care may be useful. Symbiotics may be administered both preoperatively and postoperatively to support the gut microbiota before surgery and aid in recovery afterward^4^ ^,^ ^10^. Most studies that report benefits used symbiotics in a range of 5-10 days of prescription, and a wide arrange in number and sort of probiotic bacteria is recommended^4^ ^,^ ^10^ ^,^ ^50^.

### How to manage pain perioperatively?

Pain management in ERPs involves a multimodal approach that prioritizes patient comfort while minimizing the use of opioids to avoid associated side effects. The goal is to provide effective pain relief, enhance recovery, and reduce the risk of complications^30^.

Preoperatively, the surgical team, especially the anesthesiologist, should administer analgesic medications before surgery to preemptively address pain and modulate the body’s response to surgical stress^31^. Regional anesthesia techniques, such as epidurals or peripheral nerve blocks, to provide targeted pain relief and reduce the need for systemic opioids are highly suggested^19^ ^,^ ^42^ ^,^ ^43^. A strategy to minimize intraoperative opioid use is recommended^43^ ^,^ ^56^. This may involve the use of non-opioid analgesics, such as dipyrone and nonsteroidal anti-inflammatory drugs (trometamol cetorolaco, for example), and continuation of regional anesthesia when applicable^19^.

This position paper also recommends the use of local anesthetics at the surgical site to reduce pain and minimize the use of other types of analgesia^30^. In addition, to use the anesthesiologist’s expertise to continue the multimodal analgesic approach into the postoperative period, combining medications with different mechanisms of action to address pain from various angles. In this context, we employ opioid-sparing protocols, focusing on minimizing opioid use and utilizing alternative analgesic agents^35^ ^,^ ^36^.

## CONCLUSIONS

This position paper of the Brazilian College of Digestive Surgery strongly emphasizes that the implementation of ERPs in digestive surgery represents a paradigm shift in perioperative care, transcending traditional practices and embracing an intelligent approach to patient well-being. This review has explored the multifaceted components that define an ERP, especially the ERAS and ACERTO protocols, emphasizing the integration of evidence-based interventions across the preoperative, intraoperative, and postoperative phases. ERPs in digestive surgery can benefit not only the patient but also reduce costs in digestive surgery^2^ ^,^ ^45^. As the landscape of perioperative care continues to evolve, ongoing research and innovation will shape the future of ERPs.

Collaborative efforts between multidisciplinary teams, including surgeons, anesthesiologists, nurses, dieticians, physiotherapists, and other healthcare professionals, are crucial for successful ERPs implementation. By fostering a culture of continuous improvement and staying abreast of emerging evidence, healthcare providers can ensure that ERPs remain dynamic and responsive to evolving patient needs in their institution. It is also vital to have a hospital early discharge plan in mind, which should be done during the patient’s hospital stay, identifying potential discharge needs and constraints as soon as possible. Surgeons should involve a multidisciplinary team, including physicians, nurses, social workers, therapists, the family of the patient, and other relevant healthcare professionals in the discharge planning process.

In summary, this article delved into the principles, components, and outcomes of ERPs in digestive surgery, highlighting their transformative impact on postoperative recovery. By embracing ERPs, healthcare institutions improve patient outcomes besides contributing to a paradigm of care that prioritizes individualized and evidence-based interventions, setting a new standard for surgical excellence in the modern era.
